# Estimation of the nitrogen content of potato plants based on morphological parameters and visible light vegetation indices

**DOI:** 10.3389/fpls.2022.1012070

**Published:** 2022-10-18

**Authors:** Yiguang Fan, Haikuan Feng, Xiuliang Jin, Jibo Yue, Yang Liu, Zhenhai Li, Zhihang Feng, Xiaoyu Song, Guijun Yang

**Affiliations:** ^1^ Key Laboratory of Quantitative Remote Sensing in Agriculture of Ministry of Agriculture and Rural Affairs, Information Technology Research Center, Beijing Academy of Agriculture and Forestry Sciences, Beijing, China; ^2^ School of Geographic, Liaoning Technical University, Fuxin, China; ^3^ College of Agriculture, Nanjing Agricultural University, Nanjing, China; ^4^ Key Laboratory of Crop Physiology and Ecology, Ministry of Agriculture, Institute of Crop Sciences, Chinese Academy of Agricultural Sciences, Beijing, China; ^5^ College of Information and Management Science, Henan Agricultural University, Zhengzhou, China; ^6^ Key Lab of Smart Agriculture System, Ministry of Education, China Agricultural University, Beijing, China; ^7^ College of Geomatics, Shandong University of Science and Technology, Qingdao, China; ^8^ School of Computer and Information Technology, Beijing Jiaotong University, Beijing, China

**Keywords:** potato, plant nitrogen content, UAV, vegetation indices, morphological parameters

## Abstract

Plant nitrogen content (PNC) is an important indicator to characterize the nitrogen nutrition status of crops, and quickly and efficiently obtaining the PNC information aids in fertilization management and decision-making in modern precision agriculture. This study aimed to explore the potential to improve the accuracy of estimating PNC during critical growth periods of potato by combining the visible light vegetation indices (VIs) and morphological parameters (MPs) obtained from an inexpensive UAV digital camera. First, the visible light VIs and three types of MPs, including the plant height (*H*), canopy coverage (CC) and canopy volume (CV), were extracted from digital images of the potato tuber formation stage (S1), tuber growth stage (S2), and starch accumulation stage (S3). Then, the correlations of VIs and MPs with the PNC were analyzed for each growth stage, and the performance of VIs and MPs in estimating PNC was explored. Finally, three methods, multiple linear regression (MLR), k-nearest neighbors, and random forest, were used to explore the effect of MPs on the estimation of potato PNC using VIs. The results showed that (i) the values of potato *H* and CC extracted based on UAV digital images were accurate, and the accuracy of the pre-growth stages was higher than that of the late growth stage. (ii) The estimation of potato PNC by visible light VIs was feasible, but the accuracy required further improvement. (iii) As the growing season progressed, the correlation between MPs and PNC gradually decreased, and it became more difficult to estimate the PNC. (iv) Compared with individual MP, multi-MPs can more accurately reflect the morphological structure of the crop and can further improve the accuracy of estimating PNC. (v) Visible light VIs combined with MPs improved the accuracy of estimating PNC, with the highest accuracy of the models constructed using the MLR method (S1: *R* ^2 ^= 0.79, RMSE=0.27, NRMSE=8.19%; S2:*R* ^2 ^= 0.80, RMSE=0.27, NRMSE=8.11%; S3: *R* ^2 ^= 0.76, RMSE=0.26, NRMSE=8.63%). The results showed that the combination of visible light VIs and morphological information obtained by a UAV digital camera could provide a feasible method for monitoring crop growth and plant nitrogen status.

## 1 Introduction

Worldwide demand for food has increased dramatically owing to the constraints of arable land area and the increasing global population. Some studies suggest that the yield of agricultural systems must double by 2050 to meet the growing food demand of the worldwide population (White et al., 2012; Holman et al., 2016). Staple crops, such as rice (*Oryza sativa*), maize (*Zea mays*), and wheat (*Triticum aestivum*), have a limited scope for increasing yields and high requirements for irrigation systems, which results in high production costs and low potential. Alternatively, potato (*Solanum tuberosum*) is becoming increasingly important in ensuring global food security as the fourth largest food crop with a short growth cycle and the ability to adapt to the environment (Liu et al., 2022). In recent years, the excessive application of nitrogen (N) fertilizer to aggressively maximize potato yields in some regions has reduced the efficiency of N fertilizer use, resulting in increased production costs and wasted resources and triggering potential environmental risks (Nayak et al., 2015). Therefore, scientific N fertilizer management is a vital issue that needs to be addressed for the healthy and sustainable development of the potato industry.

Plant nitrogen content (PNC) is an important indicator that is used to characterize the nitrogen nutritional status of crops. Quickly and efficiently obtaining the PNC information of crops is highly significant for evaluating crop growth and scientifically applying N fertilizers (Fu et al., 2021a). Although traditional field surveys and destructive sampling methods can obtain more accurate PNC information, they are time-consuming and inefficient. Therefore, they cannot meet the current development needs of large-scale, rapid, and efficient monitoring of crop growth conditions in precision agriculture (Yue et al., 2019; Peter et al., 2021; Li et al., 2022). In recent years, the rapid development of remote sensing technology has provided a new option for the efficient, non-destructive, and real-time monitoring of the PNC status of crops.

Compared with ground and satellite remote sensing techniques, unmanned aerial vehicle (UAV) imaging technology can obtain higher temporal and spatial resolution and is more suitable for crop growth monitoring and estimating physicochemical parameters at the farm scale (Yue et al., 2021a). A substantial amount of research has been conducted on UAV imaging technology to monitor the N nutrition status of crops. For example, Feng et al. (Feng et al., 2016) combined the normalized difference red-edge index (NDRE) and floating-position water band index (FWBI) to construct a new vegetation index – the water-tolerant nitrogen index (WNI), which effectively improved the accuracy of estimating the N content in winter wheat leaves. Wang et al. (Wang et al., 2012) showed that the three-bands vegetation index with wavelengths of 423 mm, 703 mm, and 924 mm was significantly better than the two-bands vegetation index in monitoring the nutrient status of N in rice. Xu et al. (Xu et al., 2021) fused multi-source sensors information to construct coverage-adjusted spectral indices (CASIs) to estimate the content of leaf N of maize in three reproductive stages. The results showed that the CASIs outperformed conventional spectral indices. These studies showed that vegetation indices (VIs) can effectively characterize the N nutrient status of crops compared with the traditional methods, which will aid in the efficient management of nitrogen fertilization in the field. However, most of these indices contain wavelengths other than visible light, such as red-edge and near-infrared bands and require the integration of narrow-band reflectance (He et al., 2016; Lu et al., 2021). Simultaneously, the sensors used to acquire these bands, such as hyperspectral and multispectral, are expensive. They have complicated data processing processes that increase the cost of agricultural production and limit their large-scale application in agricultural remote sensing.

In contrast to sensors, such as hyperspectral and multispectral, high-definition digital cameras are inexpensive and have high spatial resolution, simple data processing, and stable performance (Li et al., 2020; Lu et al., 2021). The use of inexpensive digital cameras to monitor the N nutrition status of crops has gradually become favored by many researchers (Putra and Soni, 2018; Putra and Soni, 2020). However, owing to the few wavelength channels of digital camera sensors, the red-edge and near-infrared bands closely related to the crop canopy structure cannot be obtained (Prey et al., 2018; Jin et al., 2021). Thus, there are certain limitations in monitoring crop N nutrition using VIs that have only been constructed with visible light (Wang et al., 2014).

Morphological parameters (MPs), such as plant height (*H*) and canopy cover (CC), are direct expressions of crop growth and nutritional status, as well as a comprehensive reflection of N metabolism in the crop (Yue et al., 2021b; Li et al., 2022). Similar to the red-edge and near-infrared bands, MPs can provide structural information closely related to crop growth. The combination of *H* and CC extracted from UAV digital images has been shown to significantly improve the accuracy of VIs in estimating crop growth parameters, such as yield, biomass, and the leaf area index (Lu et al., 2019; Wan et al., 2020; Qiao et al., 2022; Shu et al., 2022). Furthermore, most of the growth parameters described above are closely related to the N nutrient status of crops. Therefore, there should also be some connection between MPs and the N status of crops. However, whether the structural information provided by MPs can be used to monitor the nitrogen status of crops remains to be further investigated. The high spatial resolution of UAV digital cameras makes them unique at extracting crop MPs, which provides a new concept to effectively monitor the N nutrient status of crops using inexpensive digital cameras.

However, there are differences in the ability of different MPs to characterize the growth status of crops. For example, *H* and CC reflect the morphological information of crops in vertical and canopy structures, respectively (Bendig et al., 2015; Maimaitijiang et al., 2019). With the advance in growth period, the values of *H* and CC may tend to be stable and no longer change significantly (Tilly et al., 2015). The use of only a single MP may not be able to accurately reflect the dynamic changes of crop growth (Niu et al., 2019; Fu et al., 2021b). Thus, this study calculated the canopy volume (CV) of crops based on the product of *H* and CC, explored the relationship between multiple MPs (*H*, CC and CV) and the potato PNC, and used the three methods of multiple linear regression (MLR), k-nearest neighbors (KNN) and random forest (RF) to explore the performance of MPs and MPs combined with visible light VIs to estimate the potato PNC with the goal of providing a new method to effectively monitor the N status of crops with an inexpensive digital camera.

In summary, this study utilized potato as the research object and explored the potential of MPs extracted by inexpensive digital camera and those combined with visible light VIs to estimate the potato PNC to provide technical support for the scientific and precise management of potato N nutrition. The specific goals of this study were to: (1) evaluate the accuracy of extraction of potato MPs by a UAV digital camera; (2) compare the performance of different MPs and the combination of multi-MPs to estimate the potato PNC; and (3) investigate the effect of MPs on the estimation of potato PNC by visible light VIs and evaluate the potential of combining the two to improve the accuracy of estimating PNC.

## 2 Experiment and methods

### 2.1 Experimental design

**Figure 1:**
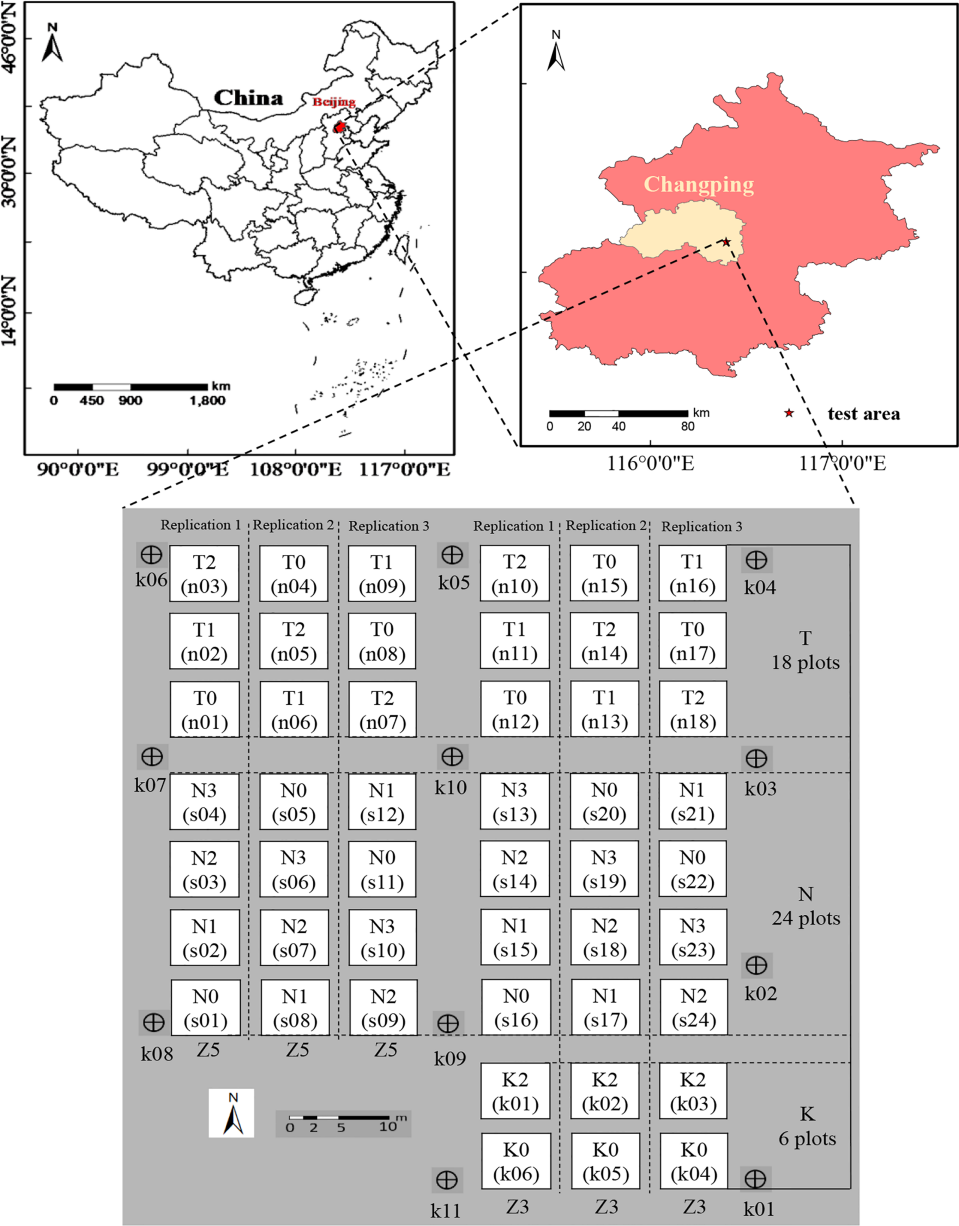
Potato field location and experimental design.

The experiment was conducted from April to July 2019 at the National Precision Agriculture Experiment Station (40°10´N, 116°26´E), Changping District, Beijing, China. The average altitude is 36 m, and the climate type is a warm temperate semi-humid continental monsoon. Potato seed tubers were sown on 28 March 2019 and harvested on 9 July 2019. The experimental area was divided into the density experimental area (T plots), N experimental area (N plots) and potassium fertilizer experimental area (K plots) to increase the spatial difference of potato growth (Liu et al., 2022). Among them, three levels were established in the density test area, including 60,000 plants/hm^2^ (T0), 72,000 plants/hm^2^ (T1), and 84,000 plants/hm^2^ (T2). Two early maturing potato varieties Zhongshu 5 (Z5) and Zhongshu 3 (Z3) were the two varieties under each density treatment and the experiments were conducted in triplicate with a total of 18 plots. Four levels of N were established in the N test area, including 0 kg/hm^2^ urea (N0), 244.65 kg/hm^2^ urea (N1), 489.15 kg/hm^2^ urea (N2, normal treatment, 15 kg of pure N), and 733.50 kg/hm^2^ urea (N3). The same two varieties (Z5 and Z3) were under each N treatment and three replicates for a total of 24 plots. Three levels were established in the potassium fertilizer test area, including 0 kg/hm^2^ potassium fertilizer (K0), 970.50 kg/hm^2^ potassium fertilizer (K1, the planting density and nitrogen test area received the K1 treatment), and 1,941 kg/hm^2^ potassium fertilizer (K2)under one variety treatment (Z3), which was repeated three times for a total of six plots. Both N and K plots were treated under T1 density. There were 48 test plots in total, and the area of a single plot was 32.5 m^2^. A total of 11 ground control points (k01~k11) were evenly buried around the test area to accurately obtain the spatial location of the test area and reduce the influence of the positional deviation of each growth period on the test results, and the three-dimensionality (3-D) of each ground control point (GCP) was measured by high-precision GPS. The location of the test field and the details of the test plan are shown in **Figure 1**.

### 2.2 UAV digital images acquisition and pre-processing

UAV flight operations were conducted on April 20, May 28, June 10, and June 20, 2019, to obtain digital images of the potato bare soil stage, tuber formation stage (S1), tuber growth stage (S2), and starch accumulation stage (S3). A DJI Genie 4Pro UAV (DJI Group, Ltd. Shenzhen, China) was used as the remote sensing system platform, and it carried a COMS sensor with 20 million effective pixels and three wavelength channels, including Red (R), Green (G), and Blue (B). In addition, the system was equipped with a position and orientation system (POS) to record the position and spatial altitude of the camera center during data acquisition. The UAV flights were operated in clear, cloudless, and calm weather conditions between 11 a.m. and 1 p.m. local time. The flight altitude was established to 20 m; the overlap rate of heading and collateral direction was 85%, and the spatial resolution of the images obtained was approximately 0.86 cm.

The digital images were pre-processed using Agisoft PhotoScan Professional software (Agisoft, LLC, St. Petersburg, Russia). The specific processing flow started with the digital images with POS data, and the 3-D coordinates of GCPs in the potato bare soil period and each growth period were imported into the software. Images with abnormal attitude angles were removed, and the images were initially aligned. The spatial position and attitude of the photography center at the moment of image acquisition was restored. Secondly, the images were topographically corrected based on the 3-D coordinates of the GCPs to further optimize the spatial attitude and position of the images and generate a sparse point cloud with precise spatial information attributes in the flight area. Next, the dense point cloud of the flight area was constructed to generate a spatial grid and texture information. Finally, the digital orthophoto map (DOM) and digital surface model (DSM) of the test area were generated.

### 2.3 Ground data acquisition

The ground data collection was simultaneously conducted with the UAV flight operation and primarily included digital ground photos and measurements of the plant height and PNC at each growth stage. The digital ground photos were obtained by first placing a 1.3 m × 1.3 m white box (perpendicular and parallel to the test crop rows) randomly in each test plot and then using a Canon G16 digital camera to horizontally photograph at 2 m directly above the white box to obtain ground digital photo of each test plot to extract the ground coverage. The plant height was measured by selecting four representative plants in each plot and measuring the distance from the base of the stem to the tip of the leaf and recording it. Finally, the average height of the four plants was considered to be the measured plant height of the plot. The potato PNC was measured by selecting three representative plants in each plot, separating the stems and leaves, and then killing them at 105°C for 0.5 h. The plants were then dried at 80°C to a constant mass and weighed. The N contents of the stem and leaf parts were measured separately using a Kjeldahl nitrogen analyzer. Finally, the PNC was calculated based on the dry mass and nitrogen content of the samples (Fu et al., 2020). The statistical analysis of the measured plant height and PNC at each growth stage is shown in **Table 1**.

**Table 1:** Statistical analysis of plant height and nitrogen content of potato in different growth stages.

Growth stages	Crop parameters	Max	Min	Mean	Standard deviation	Coefficient of variation (%)
S1	*H*	40.50	20.38	30.29	4.76	15.71
	PNC	4.50	2.09	3.21	0.61	19.03
S2	*H*	40.88	20.42	27.72	5.20	18.75
	PNC	4.00	1.61	2.69	0.58	21.41
S3	*H*	40.35	15.12	25.78	5.15	19.97
	PNC/%	3.74	1.86	2.94	0.46	15.86

S1, tuber formation stage; S2, tuber growth stage; S3, starch accumulation stage.

### 2.4 Vegetation index selection

Based on the existing research results in which visible light VIs were used to monitor crop N status, 10 VIs with potential performance for estimating the potato PNC were selected for follow-up studies as shown in **Table 2**. Among them, R, G, and B represent the digital number (DN) values of the red, green, and blue channels, respectively, and r, g, and b were calculated from equations(1)–(3), which represent the digital numbers of the normalized R, G, and B, respectively.


(1) $$\text{r=}\frac{\text{R}}{\text{R+G+B}}$$

(2) $$\text{g=}\frac{\text{G}}{\text{R+G+B}}$$

(3) $$\text{b=}\frac{\text{B}}{\text{R+G+B}}$$

**Table 2:** Visible light vegetation indices related to nitrogen.

Visible light vegetation indices	Definition	Reference
R	R	/
G	G	/
B	B	/
GRRI	r/g	(Lu et al., 2021b)
GLA	(2*g-r+b)/(2*g+r+b)	(Yue et al., 2021b)
GLI	(2*g-r-b)/(2*g+r+b)	(Yue et al., 2018; Zhou et al., 2018)
GRVI	(g-r)/(g+r)	(Bendig et al., 2015)
VARI	(g-r)/(g+r-b)	(Maimaitijiang et al., 2019)
EXG	2*g-b-r	(Fu et al., 2021a)
NDI	(r-g)/(r+g+0.01)	(Meyer and Neto, 2008)

### 2.5 Extraction of morphological parameters

The acquisition of *H* in the different growth stages of potato was primarily determined by the difference between the DSM in each growth stage and that in the bare soil stage. The specific methods first included obtaining the high-definition digital images of the experimental field in the bare soil period and combining them with the 3-D coordinates of GCPs. The DSM of this period, namely DSM_0_, was generated, which was used as the reference plane for the subsequent *H* extraction. Secondly, based on the digital images of different growth stages of potato, combined with GCPs, the DSM of the corresponding growth stage, namely DSM*_i_* (*i*=1, 2, 3, denoted S1, S2, and S3, respectively) was generated. DSM*_i_* was then differentiated from DSM_0_ (Equation 4) to obtain the crop height models for the corresponding growth periods. Finally, the average plant height of each plot was extracted using ENVI 5.3 software (L3Harris Geospatial, Boulder, CO, USA) and the vector data of each experimental plot was used to obtain the *H* of each plot.


(4) $$\begin{array}{ll} H_{i}={\text{DSM}}_{i}-{\text{DSM}}_{0} & \left(i=1,2.3\right) \end{array}$$

This study extracted the CC of potato in each growth period based on the ground digital photos and UAV digital images, respectively, and used the results extracted from the ground digital photos as the CC measured values to verify the results extracted by the UAV (Li et al., 2005; Meyer and Neto, 2008). Among them, the vegetation coverage extraction algorithm (VCEA) based on ground digital photos derived the concepts of CC extraction from several studies (Gitelson et al., 2003; Meyer and Neto, 2008) and optimized some of them. The basic process first involved transforming the test area based on the Hue-Saturation-Intensity (HSI) color space, and secondly, using the Excess Green Vegetation Index (EXG) to conduct green vegetation processing on the results of HSI processing. The soil background and weed noise were removed using the maximum interclass variance threshold and morphological threshold. Finally, the ratio of the number of pixels of vegetation in each plot to the total number of pixels in the plot was calculated, which is the measured CC value of the plot. The basic process of CC extraction based on UAV digital images included first processing the DOM of each growth period using the EXG index. The threshold value of vegetation and soil was then obtained using the bimodal method, and the number of pixels of vegetation and soil in each plot was obtained using the banding operation. Finally, the ratio of number of pixels of vegetation to the total number of pixels in each plot was calculated, which was the CC value of that plot based on UAV extraction.

In this study, the product of extracted *H* and CC was defined as the canopy volume (Qiao et al., 2022) to explore the association of multiple MPs with the potato PNC, and the CV was calculated as shown in Equation 5.


(5) CV=CC*H

### 2.6 Model building and evaluation

A total of 48 sets of data were obtained in each growth period of potato. To enhance the reliability of the experimental conclusions, the models were constructed with repetitions 1 and 3 (32) as the training set, and the data of repetition 2 (16) was used to validate the models. The methods used to build the potato PNC estimation models included MLR, KNN, and RF. Among them, MLR is an effective linear regression method, which is often used to describe the linear relationship between multiple independent variables and dependent variables. KNN is a mature machine learning algorithm that can determine the regression values of the samples to be tested based on the features of the *k* most similar samples in the feature space. RF is a supervised ensemble learning algorithm. It trains input samples to generate a decision tree training set based on bootstrap resampling technology, and then integrates the results of each decision tree to output the predicted target value. In this study, the coefficient of determination (*R*²), root mean square error (RMSE), and normalized root mean square error (NRMSE) were used to evaluate the accuracy and stability of the models.

## 3 Results and analysis

### 3.1 Potato plant height extraction

**Figure 2:**
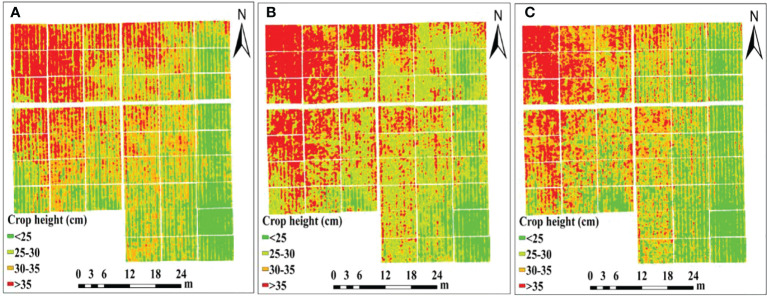
Crop height models of potato at **(A)** S1, **(B)** S2, **(C)** S3.

The crop height models based on UAV digital images can visually represent the spatial distribution of potato plant height at different growth stages, which helps to monitor the growth of potato plants, and explore the effects of varying treatment factors on the height of potato plants. In this study, the DSM of different growth stages of potato and that of the bare soil stage were calculated to obtain the crop height models of corresponding growth stages, and the results are shown in **Figure 2**. It is apparent that the height of potato plant in each growth period was generally high in the west and low in the east. Among them, there were differences in the plant height between different varieties, nutrient and density treatments, and the difference in plant height of the different varieties of potato was the most obvious.

**Figure 3:**
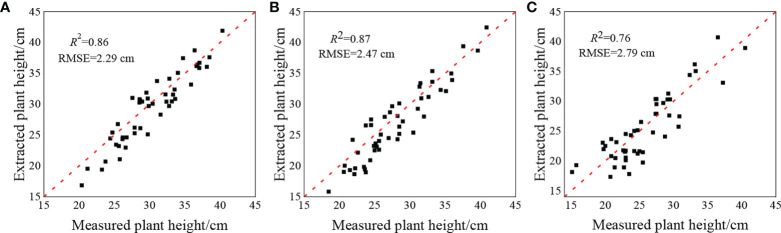
Contrastive analysis of plant height extracted from potato and measured plant height at **(A)** S1, **(B)** S2, **(C)** S3.

To verify the accuracy of *H* extraction based on the DSM, the measured plant height and extracted plant height were compared and analyzed for the three reproductive stages, and the results are shown in **Figure 3**. The coefficients of determination of the extracted plant height and the measured plant height in the three growth stages were 0.86, 0.87 and 0.76, respectively, and the RMSE were 2.29 cm, 2.47 cm, and 2.79 cm, respectively. These values indicated that the plant height extraction based on DSM had higher precision, and the extraction precision of potato in the early stages of growth was higher than that in the later stage of growth.

### 3.2 Potato coverage extraction

**Figure 4:**
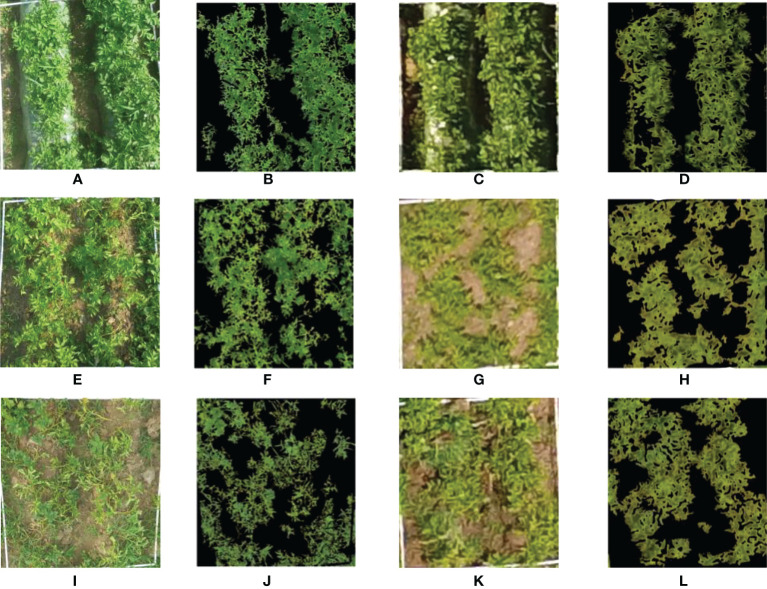
Comparison of two methods for extracting potato coverage.**(A, E, I)** Ground digital photos of the potato plants at S1-S3; **(B, F, J)** The effect of potato CC extraction using ground digital photo at S1-S3; **(C, G, K)** UAV digital images of potato plants at S1-S3; **(D, H, L)** The effect of potato CC extraction using UAV digital image at S1-S3.

The ground digital photos and UAV digital images of three growth stages of the potato plants were processed by VCEA and EXG index bimodal threshold methods, respectively, and the CC values of each growth stage that were measured and extracted were obtained. The use of plot s20 as an example in **Figure 4** compares the results of CC extraction by the two methods. The digital ground photos and the UAV digital images showed slight differences in potato canopy morphology. Among them, the digital ground photos more clearly reflected the interplant interlacing state of potato, which was more conducive to the extraction of potato canopy cover. UAV digital images can also better distinguish potato plants from soil background, and the CC values extracted based on the two methods can effectively reflect the potato canopy cover in general.

**Figure 5:**
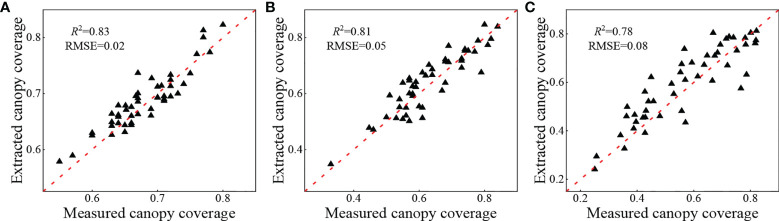
Comparative analysis of potato extraction coverage and measured coverage at **(A)** S1, **(B)** S2, **(C)** S3.

The CC that was extracted using the two methods for the three growth stages was compared and analyzed to quantitatively evaluate the accuracy of CC extraction based on the UAV digital images. The results are shown in **Figure 5**. The CC values of the S1 were primarily concentrated between 0.6 and 0.8, while the CC values of the S2 and S3 were primarily concentrated between 0.4 and 0.8. The coefficients of determination of the fit between the extracted CC and the measured CC in the S1, S2, and S3 were 0.83, 0.81, and 0.78, respectively. The RMSE were 0.02, 0.05, and 0.08, respectively, indicating that the accuracy of potato canopy cover extracted based on digital images is reliable and can be used to estimate crop physical and chemical parameters.

### 3.3 Correlation analysis between the parameters and PNC

**Figure 6:**
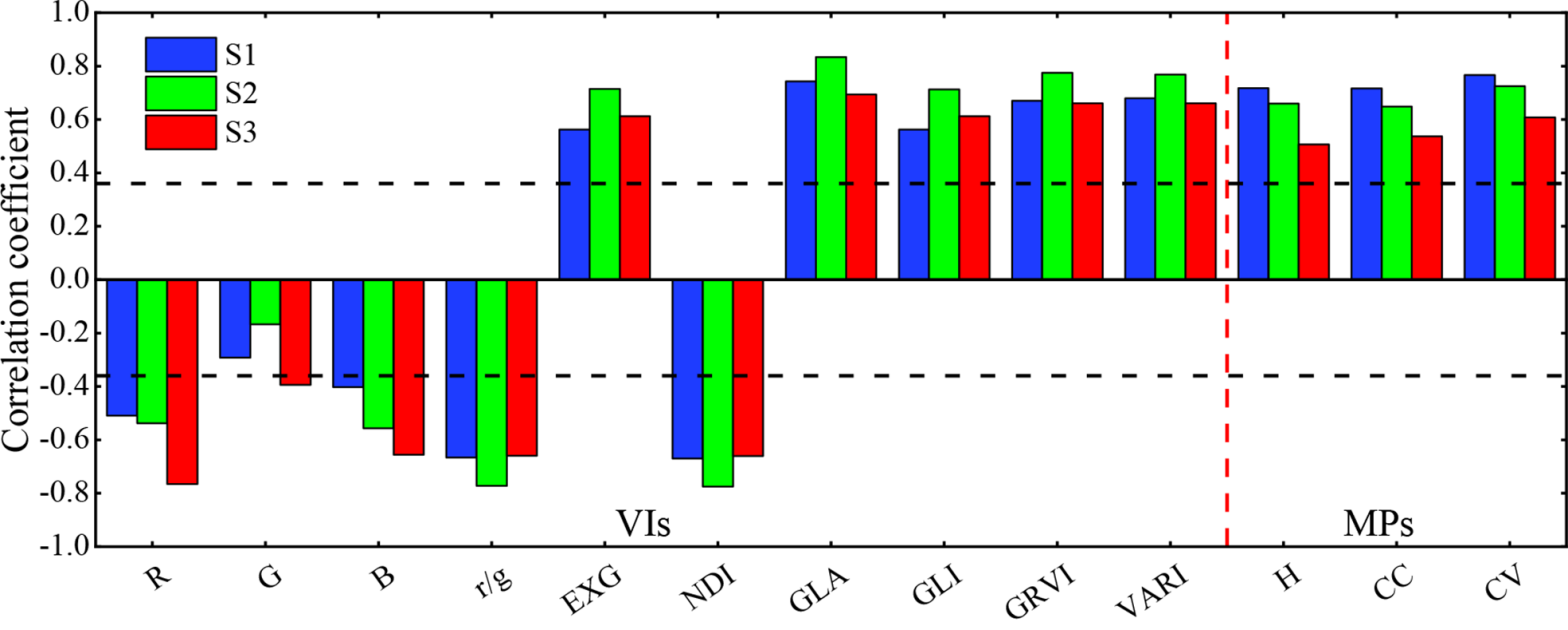
Correlation analysis results of each parameter and potato PNC. Note: the horizontal dashed line indicates a significance level of 0.01.

Correlation analysis was performed between the visible light VIs and MPs obtained in each growth period of potato and PNC, and the results are shown in **Figure 6**. The correlation between most of the VIs and PNC tended to increase and then decrease from the S1 to S3. Among them, all the VIs except G reached a significance level of 0.01 for the correlation with the PNC during the S1 and S2, and the absolute values of correlation coefficients ranged from 0.40 to 0.74 and 0.54 to 0.83, respectively. All the VIs reached a significance level of 0.01 for the correlation with the PNC during the S3, and the absolute values of correlation coefficients ranged from 0.40 to 0.69. Unlike visible light VIs, the correlation between MPs and PNC gradually decreased as the growing season progressed. Nevertheless, the correlation between all the MPs and PNC a significance level of 0.01 in all three growth stages. Furthermore, the correlations of CV were higher than those of *H* and CC, and the absolute values of the correlation coefficients were 0.77, 0.63, and 0.65 for the three growth stages S1, S2, and S3, respectively. Compared with the visible light VIs, the correlations between MPs and PNC in the three growth stages of potato were comparable to those of most of the visible light VIs, indicating that the association between MPs and PNC is stronger and that it is practical to monitor the potato PNC based on the MPs extracted from inexpensive digital cameras.

### 3.4 Estimation of the Potato PNC

#### 3.4.1 Estimation of the potato PNC by visible light VIs

In this study, the top five VIs with higher correlations in each growth period were selected, and the three methods MLR, KNN and RF were used to construct estimation models for the potato PNC. The results are shown in **Table 3**. The *R* ^2^ of potato PNC estimation models constructed using the three methods > 0.5 for all three growth periods, indicating that the selected VIs could reflect the potato PNC status some extent. Thus, it was feasible to monitor the potato PNC status based on visible light VIs during the critical growth periods. A comparison of the results of the three reproductive stages indicated that the estimations of the S1 and S2 were clearly better than that of the S3. From the modeling and validation results, it can be seen that the potato PNC estimation models constructed by the three methods show similar *R* ^2^, RMSE, and NRMSE.

**Table 3:** Visible light vegetation indices estimation of potato PNC.

Growth stages	Dataset	MLR			KNN			RF		
		*R^2^*	RMSE/%	NRMSE/%	*R^2^*	RMSE/%	NRMSE/%	*R^2^*	RMSE/%	NRMSE/%
S1	Cali	0.75	0.31	9.76	0.72	0.33	10.45	0.75	0.31	9.95
	Vali	0.75	0.30	8.99	0.72	0.34	10.32	0.73	0.36	10.67
S2	Cali	0.74	0.27	10.43	0.69	0.31	11.44	0.73	0.28	10.70
	Vali	0.78	0.32	11.23	0.62	0.42	14.77	0.73	0.38	13.52
S3	Cali	0.69	0.24	8.42	0.62	0.27	9.37	0.68	0.26	8.83
	Vali	0.72	0.29	10.12	0.64	0.32	8.90	0.64	0.31	10.55

#### 3.4.2 MPs to estimate the potato PNC

**Figure 7:**
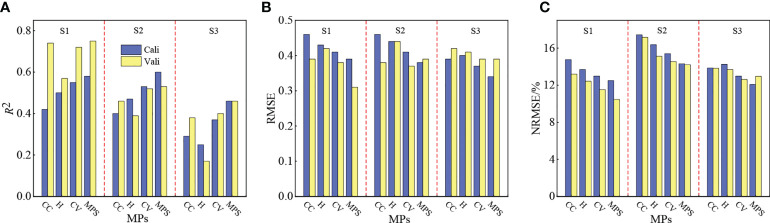
Estimation of the potato PNC effect by morphological parameters at **(A)** *R* ^2^, **(B)** RMSE, **(C)** NRMSE.

To investigate the association between MPs and the PNC, this study constructed estimation models of the potato PNC based on extracted *H*, CC, and constructed CV. The three were combined to investigate the effect of multiple MPs in estimating the potato PNC. The results are shown in **Figure 7**. The estimation of PNC by single or multiple MPs was better in the S1 and worse in the S3, whereas *H* and CC were the worst in the S3 (*R^2^* < 0.4). The models constructed by CV had a higher *R* ^2^, lower RMSE, and NRMSE and better estimation of PNC than those of *H* and CC. The combination of multiple MPs effectively improved the accuracy of PNC estimation at all the growth stages of potato compared with the models constructed with single MPs. However, compared with visible light VIs (**Table 3**), the estimation effect of MPs requires further improvement.

#### 3.4.3 Estimation of the potato PNC by combining visible light VIs and MPs

**Figure 8:**
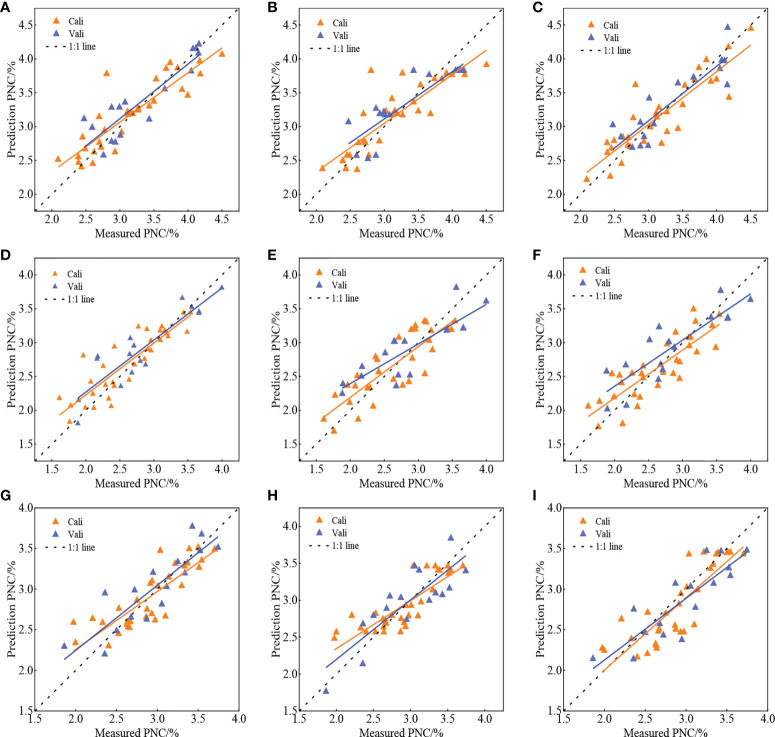
Validation effect of estimation of potato PNC based on fusion characteristics at each growth stage.**(A, D, G)** MLR, S1-S3; **(B, E, H)** RF, S1-S3; **(C, F, I)** KNN, S1-S3.

To investigate the effect of inexpensive digital camera extraction of MPs on the visible light VIs to estimate the potato PNC, this study constructed the PNC estimation models for the three potato growth stages using three methods MLR, KNN, and RF based on the selected VIs and extracted MPs in Section 2.5. The results are shown in **Table 4**. The models used to estimate the PNC were constructed using three methods with visible light VIs combined with MPs. They were also more effective at estimating the S1 and S2 than the S3 for potato at all stages of growth. Combined with **Tables 3**, **4**, it is apparent that during the same growth period, the *R* ^2^ of the models constructed by VIs combined with MPs increased compared with a single model variable. In addition, the RMSE and NRMSE decreased substantially, indicating that the addition of MPs improved the accuracy of visible light VIs in estimating the PNC. Comparing the estimation results of different methods in each growth stage, it can be seen that the three methods have achieved promising results. The scatter plot of the predicted and measured PNC values for each growth period are shown in **Figure 8**. The predicted and measured PNC values obtained by the three methods were mostly uniformly distributed around the 1:1 line for each potato growth period, indicating that the overall effect of estimating the PNC based on visible light VIs and MPs is suitable.

**Table 4:** Estimation of the potato PNC by visible light vegetation indices combined with morphological parameters.

Growth stages	Data set	MLR			KNN			RF		
		*R^2^*	RMSE/%	NRMSE/%	*R^2^*	RMSE/%	NRMSE/%	*R^2^*	RMSE/%	NRMSE/%
S1	Cali	0.78	0.29	9.07	0.75	0.31	9.78	0.78	0.29	9.20
	Vali	0.79	0.27	8.19	0.76	0.29	8.84	0.81	0.35	10.58
S2	Cali	0.76	0.27	10.36	0.71	0.29	10.99	0.73	0.29	10.95
	Vali	0.80	0.27	8.11	0.70	0.36	12.73	0.75	0.34	11.90
S3	Cali	0.71	0.24	8.13	0.70	0.25	8.39	0.72	0.26	9.21
	Vali	0.76	0.26	8.63	0.70	0.29	9.65	0.73	0.28	9.53

## 4 Discussion

### 4.1 Extraction of the morphological parameters

The highly accurate extraction of the potato *H* and CC at all growth stages is essential to explore the potential of MPs in combination with the visible light VIs to estimate the potato PNC. UAV remote sensing platforms are advantageous because of their operational flexibility and high spatial and temporal resolution; thus, they have more significant advantages in extracting crop MPs (Holman et al., 2016; Li et al., 2016; Niu et al., 2019; Johnson et al., 2020). In this study, based on the crop height models of potato, the plant height was extracted in three growth stages, and it is apparent in **Figure 3** that the extraction of the plant height in all three growth stages of potato was highly accurate, and the accuracy of the S1 and S2 was better than that of the S3. The reason is that during the first two growth periods, the potato plants primarily grew vegetatively and reproduced, producing vigorous plants with a large area of leaf expansion. At this time, the plant height extracted was less affected by mixed pixels and was highly precise. The potato tubers were bulking and maturing during the S3, and some potato leaves began to turn yellow and shrink, and the canopy coverage was reduced. At this time, the extracted plant height was substantially affected by the soil background, which reduced the accuracy. Similar to the plant height, the accuracy of extraction of the CC also showed that the S1 and S2 could be more effectively extracted than plants in the S3. The reason for this is that the extraction of CC was less influenced by soil and weeds and more accurate during the early growth periods of potato. In contrast, there was a larger difference between the CC values extracted based on digital ground photos and those based on UAV digital images during the S3. The accuracy of extraction of CC decreased owing to the influence of a small number of field weeds and soil background. As shown in **Figures 3**, **5**, the extraction of the potato canopy MPs based on the UAV digital camera was highly accurate and more effectively reflected the growth conditions of potato, which enables their use to estimate physical and chemical parameters. In addition, measures, such as mulching and weeding, can be implemented in the field to reduce the interference of soil background and weeds and further improve the accuracy of extracting crop MPs.

### 4.2 Response of visible light VIs to the PNC

Based on the existing research results, this study selected 10 VIs that were closely linked to crop N and analyzed their correlation with the PNC in three key growth stages of potato. As shown in **Figure 6**, most of the VIs and PNC reached a significance level of 0.01 for the correlation, indicating that it is feasible to use visible light VIs to estimate the PNC. The correlation between most VIs and PNC first increased and then decreased from the S1 to S3. The effect of estimating the PNC based on visible light VIs (**Table 3**) also showed that the estimation of the early growth stages of potato were better than those at the later stage.

The reason is that the vegetative and reproductive growth were the primary factors during the early stages of potato growth. The plants grew vigorously, and the extracted VIs were less affected by mixed pixels, such as soil, so they more effectively reflected the change in PNC. In contrast, the effect of spectral saturation rendered most of the VIs less sensitive to the evolution of PNC during the late stage of potato growth, and some potato plants began to senesce and turn yellow during this period. The spectral information extracted at this time was also substantially affected by the soil background. Therefore, the accuracy of estimation of PNC by visible light VIs was lower during the S3 than during the first two reproductive stages. In addition, compared with Nigon’s result (Nigon et al., 2015) of estimating the content of N in potato leaves (*R* ^2 ^= 0.79), the accuracy of this study was lower. There are several primary reasons for this. On the one hand, Nigon used the red edge information obtained by a hyperspectral camera to obtain more spectral information related to nitrogen (Raper and Varco, 2015). Alternatively, compared with the N content of plants, changes in the canopy spectrum were more closely related to the content of crop leaf N than that of the plant, and the spectral information is more suitable for estimating the leaf N content (Zhou et al., 2018).

### 4.3 Response of morphological parameters to the PNC

The MPs of crops have been widely used to monitor crop growth parameters (Bendig et al., 2015; Stevens et al., 2020). However, the status of response of PNC to different MPs at the various crop growth stages is unclear. As shown in **Figures 6** and **7**, the correlation between all three MPs and the PNC gradually decreased as the growth period advanced, and the constructed models gradually became less accurate. The reason for this is that the potato growth was most closely related to the nutritional status during the S1, and the accuracy of extraction of each MP was higher, which more effectively reflected the changing status of PNC. During the S2, *H* and CC tended to saturate, and no longer changed significantly (Wan et al., 2020; Qiao et al., 2022), and the link between MPs and PNC weakened. The growth of potato was primarily reproductive during the S3, and the N in plant continued to transfer to the tuber. While the changes of *H* and CC were not obvious, the accuracy of extraction also became worse. The connection between MPs and PNC was weakest at this stage, and the models constructed were the least effective at estimating the PNC.

Considering that a single MP cannot finely reflect the crop growth condition, this study constructed the CV based on the extracted *H* and CC. In addition, we explored the effect of multiple MPs in estimating the potato PNC by combining *H*, CC, and CV. **Figures 6**, **7** show that the correlation between CV and PNC was higher than that of *H* and CC for all three reproductive stages of potato, and the effect of estimating PNC based on the CV was better than that based on the *H* and CC. The reason is that the PNC was composed of two parts, which included the contents of leaf N and stem N, and the CV simultaneously reflected the growth status of potato in the canopy and vertical scales, which weakened the saturation phenomenon of PNC estimated by *H* or CC. The combination of *H*, CC and CV was much more accurate at estimating the PNC than a single MP because multiple MPs can characterize the morphological changes of potato from multiple dimensions and improve the sensitivity of MPs to PNC in each period (Lu et al., 2021).

### 4.4 Effect of the MPs on the estimation of PNC from visible light VIs

Existing studies have shown that both VIs and MPs can reflect the growth and nutritional status of crops. The visible light VIs and MPs obtained by UAV digital camera enabled this study to use the three methods of MLR, KNN, and RF to explore the effect of combining the VIs and MPs to estimate the potato PNC. As shown in **Table 4**, the combination of VIs with MPs improved the accuracy of estimating PNC compared with using the VIs alone, and the accuracy of the models constructed in the three growth stages was closer to the result of Nigon. The reason is that, on the one hand, the VIs and MPs combined the nutritional information and morphological information of crops, which can better characterize the law of crop growth and enhance the connection with PNC. Alternatively, the red-edge or near-infrared band was sensitive to crop canopy structure, while *H* and CC were the primary factors that affect the crop canopy structure; the combination of multiple MPs provides similar information to the red-edge and near-infrared bands (Wang et al., 2012; Wan et al., 2020), which enhanced the link between optical VIs and PNC. Thus, the combination of visible light VIs and MPs can improve the accuracy of estimating the PNC.

### 4.5 Implications for future study

In this study, digital images of the key growth periods of the potato were obtained using a UAV digital camera, and the visible light VIs and MPs, such as *H*, CC, and CV, were extracted from them. Combined with different regression methods, the effect of different MPs and MPs combined with VIs in estimating the potato PNC were explored. The results showed that the morphological information of potato plant was closely related to the N nutrition status, and the combination of VIs and MPs could improve the accuracy of estimating the PNC, which was consistent with the existing research conclusions (Maimaitijiang et al., 2019; Shu et al., 2022). In addition, **Figures 3** and **5** showed that the *H* and CC of potato plant extracted based on the UAV digital camera were highly accurate, which could provide a favorable reference for monitoring the growth of potato. The use of MPs extracted by an inexpensive UAV digital camera combined with the visible light VIs to estimate the potato PNC not only fully utilizes the advantages of high spatial resolution of the digital camera but also avoids the possible matching error between multi-source sensors, which can provide an effective manner to estimate the physical and chemical parameters with high precision.

This study only discussed the effect of using the MPs extracted from the UAV digital images to estimate the PNC in the critical growth periods of potato at a fixed flying height. However, the accuracy of extraction of the MPs is closely related to the spatial resolution of the digital images and the flying height of the UAV. These factors should also be considered in subsequent studies. In addition, future studies should also consider using potato data from different locations and years to verify the conclusions.

## 5 Conclusions

This study developed a method to effectively estimate the growth parameters and PNC status of potato at critical growth stages based on an inexpensive UAV digital camera. First, the UAV digital camera was used to extract visible light VIs and morphological information about the potato canopy. Next, the effect of MPs and VIs combined with MPs in estimating PNC was investigated by combining various methods. Several conclusions can be drawn from these results. (1) UAV digital images can obtain potato *H* and CC information with high accuracy, which can provide a reference to assess the growth of potato plants. (2) Both visible light VIs and MPs reflect the status of potato PNC, and VIs are more closely associated with the PNC. (3) Different MPs have different effects on estimating PNC, and multiple MPs can more effectively reflect the morphological structure of crops, which can further improve the accuracy of estimating PNC. (4) Visible light VIs combined with MPs can improve the accuracy of estimating the PNC. Based on these findings, the method can provide a reference to monitor crop growth and N nutrient status using a UAV digital camera to reduce agricultural production costs and improve precision agricultural management.

## Data availability statement

The original contributions presented in the study are included in the article/supplementary material. Further inquiries can be directed to the corresponding author.

## Author contributions

HF, YF, GY, ZL, and JY designed the experiments. HF, ZF, ZL, and YL collected the PNC and UAV digital images. YF and HF analyzed the data and wrote the manuscript. XJ and HF made comments and revised the manuscript. All authors contributed to the article and approved the submitted version.

## Funding

This study was supported by the Key scientific and technological projects of Heilongjiang province (2021ZXJ05A05), the National Natural Science Foundation of China (41601346), the Platform Construction Funded Program of Beijing Academy of Agriculture and Forestry Sciences (No.PT2022-24), and the Key Field Research and Development Program of Guangdong Province (2019B020216001).

## Acknowledgments

We thanks to the National Precision Agriculture Experiment Station for providing the test site and employees. We are also grateful to Hong Chang, Huiling Long, Yang Meng and Yu Zhao who worked hard in the field and lab to provide us with valuable data.

## Conflict of interest

The authors declare that the research was conducted in the absence of any commercial or financial relationships that could be construed as a potential conflict of interest.

## Publisher’s note

All claims expressed in this article are solely those of the authors and do not necessarily represent those of their affiliated organizations, or those of the publisher, the editors and the reviewers. Any product that may be evaluated in this article, or claim that may be made by its manufacturer, is not guaranteed or endorsed by the publisher.
